# Evaluation of spatial distribution and characterization of wall shear stress in carotid sinus based on two-dimensional color Doppler imaging

**DOI:** 10.1186/s12938-018-0589-y

**Published:** 2018-10-19

**Authors:** Bo Zhang, Yuqin Ma, Fang Ding

**Affiliations:** Department of Ultrasound in Medicine, Shanghai East Hospital, Tongji University School of Medicine, Shanghai, 200120 China

**Keywords:** Wall shear stress, Intima-media thickness, Atherosclerosis, Carotid sinus

## Abstract

**Objective:**

This study aims to use a wall shear stress (WSS) quantitative analysis software to analyze and evaluate the carotid sinus WSS spatial distribution and characteristics in intima-media thickness (IMT) normal and thickening group by using two-dimensional color doppler flow imaging (CDFI) so as to assist clinicians to predict the location and risk of plaque formation.

**Methods:**

According to IMT, 50 subjects was selected as IMT thickening group and 50 subjects as IMT normal group from subjects who had a carotid ultrasound examination in Shanghai East hospital during October 2016 to October 2017. This study presents the spatial distribution of the carotid sinus WSS based on the WSS quantitative analysis software and compared the spatial distribution and characteristics of the carotid sinus WSS between IMT thickening group and IMT normal group through two- and three-dimensional WSS maps and a fused WSS image.

**Results:**

The distributional regularity of WSS in both two group was: carotid sinus < common carotid artery (CCA) < internal carotid artery (ICA) and posterior-interior wall of the carotid sinus < the anterior-lateral wall of the carotid sinus. Furthermore, the WSS of CCA, ICA, the anterior-lateral proximal wall of the carotid sinus, the anterior-lateral distal wall of the carotid sinus, the posterior-interior proximal wall of the carotid sinus, and the posterior-interior distal wall of the carotid sinus in IMT thickening group was lower than the corresponding part of IMT normal group (P < 0.05).

**Conclusion:**

In summary, this WSS quantitative analysis framework by two-dimensional CDFI can measure and reflect the carotid sinus WSS spatial distribution and characteristics more accurately and visually. As a convenient tool, it may be used for clinical prediction of the plaque formation in carotid sinus in the future.

## Background

Cardio-cerebral vascular disease is a common disease with high morbidity and mortality in China, which has gradually become is a major public health concern [1]. Cardio-cerebral vascular disease is a general term for cardiovascular disease and cerebrovascular disease [2]. Previous studies have shown that atherosclerosis and early plaque rupture are the main risk factors for myocardial infarction and cerebral ischemic stroke [3]. Intima-media is the earliest site of the occurrence and development of atherosclerosis [4]. The endothelial disfunction, foam cells accumulated in the subintima, subintimal fatty deposited becoming fatty streaks, all above result in intima-media thickening [5]. With the development of atherosclerosis, the intima-media becomes thickening into plaque formation, which is a typical sign of atherosclerosis [6]. Luminal stenosis is the late stage of atherosclerosis and sometimes need endovascular revascularization [7, 8]. Therefore, it is generally considered that intima-media thickening is an important manifestation of atherosclerosis at early stage. Intima-media thickening and plaque formation are most likely to occur in the carotid sinus [9]. Because of no specific clinical symptoms, intima-media thickness (IMT) thickening or plaque formation have a deficiency for early diagnosis and prediction of atherosclerotic related diseases [10]. In the past few decades, there has been increasing evidence showed that low level, large gradient and high oscillatory wall shear stress (WSS) can destroy the inner intima and endothelial cells and accelerate the intima-media thickening and carotid atherosclerosis [11, 12]. Furthermore, the rupture of vulnerable plaques is usually observed on the shoulder of the plaque, where it is considered to be the highest of WSS [13]. Therefore, the evaluation of hemodynamic parameters such as changes in local WSS is of great importance for predicting the location of plaque rupture. Among the existing medical imaging modalities, ultrasound, as a non-invasive imaging, is the first choice for analysis of human anatomical structure, which is widely applied to clinical study for better structural visualization when compared with magnetic resonance angiography (MRA), and computed tomography angiography (CTA) [14]. Previously, the Hagen–Poiseuille equation was the most common method to evaluate WSS reasonably and accurately [15]. But this technique could not evaluate local WSS, especially WSS near irregular intravascular plaques [16]. Therefore, Hagen–Poiseuille equation is not feasible as a clinical method to evaluate local WSS around plaque by obtaining stable vessel diameter and velocity [17]. With the development of modern ultrasound technology, such as color Doppler flow imaging (CDFI) and contrast-enhanced ultrasound, not only can average WSS be measured, but also arterial hemodynamic distribution can be measured and visualized in clinical examination [18]. This may lead to modern patterns of diagnosis for plaque and atherosclerosis. In this study, through two-dimensional CDFI, the spatial distribution and characteristics of local WSS in carotid sinus were analyzed and discussed by our proposed WSS quantitative analysis software.

## Methods

### Study participants and grouping

According to IMT, 50 subjects (IMT ≤ 0.9 mm) was selected as IMT thickening group and 50 subjects (0.9 mm < IMT ≤ 1.2 mm) as IMT normal group from subjects who had a carotid ultrasound examination by an experienced sonographer (Fang Ding) in Shanghai East hospital during October 2016 to October 2017. Exclusion criteria: severe arrhythmia, heart failure and aortic stenosis. The Ethics Committee of the Shanghai East Hospital approved the study, and written informed consent was obtained from all subjects.

### Inspection methods

The Philips IE33 system (Philips Medical Systems, Andover, MA, USA) with a L11-3 linear array transducer was used in our study. Before the carotid ultrasound examination, all subjects took a 10-min rest and no taking irritating food such as coffee and tea. The subjects took the supine position with the neck exposed and the head shifted to the opposite side. We firstly adjusted the speed range to fill the lumen with full blood flow without aliasing, and then selected a proper sampling frame range in order to keep the Doppler graphics frame frequency between 20 and 30 frames.

#### Conventional parameters of carotid ultrasound

The conventional data of carotid ultrasound was collected, such as the internal diameter of the CCA, IMT, the peak systolic velocity (PSV) and the vascular resistance index (RI). Then, four locations of carotid sinus in both two group were measured, namely the anterior–lateral proximal wall, the anterior-lateral distal wall, the posterior-interior proximal and the posterior–interior distal wall. The conventional WSS was estimated by traditional Hagen–Poiseuille formula: $\tau_{w} = \frac{{2\mu u_{m} }}{R}$ [17]. Here, $\tau_{w}$ is WSS, *μ* is blood viscosity, $u_{m}$ is the highest velocity of flow (velocity at the center of the lumen), and *R* is the inner radius of the lumen.

#### WSS quantitative analysis by CDFI

**Fig. 1:**
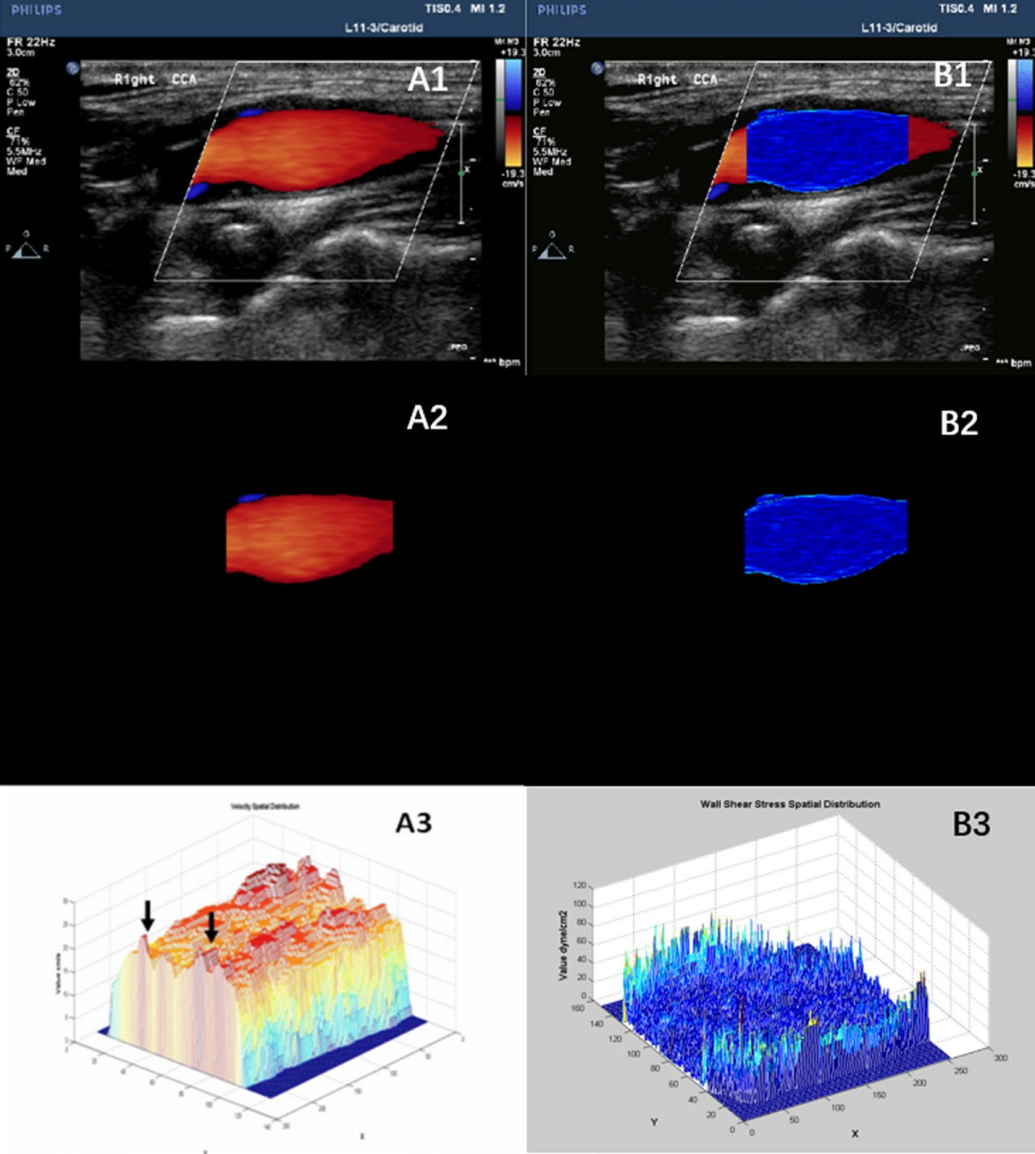
WSS spatial distribution of normal carotid sinus by WSS quantitative analysis software

**Fig. 2:**
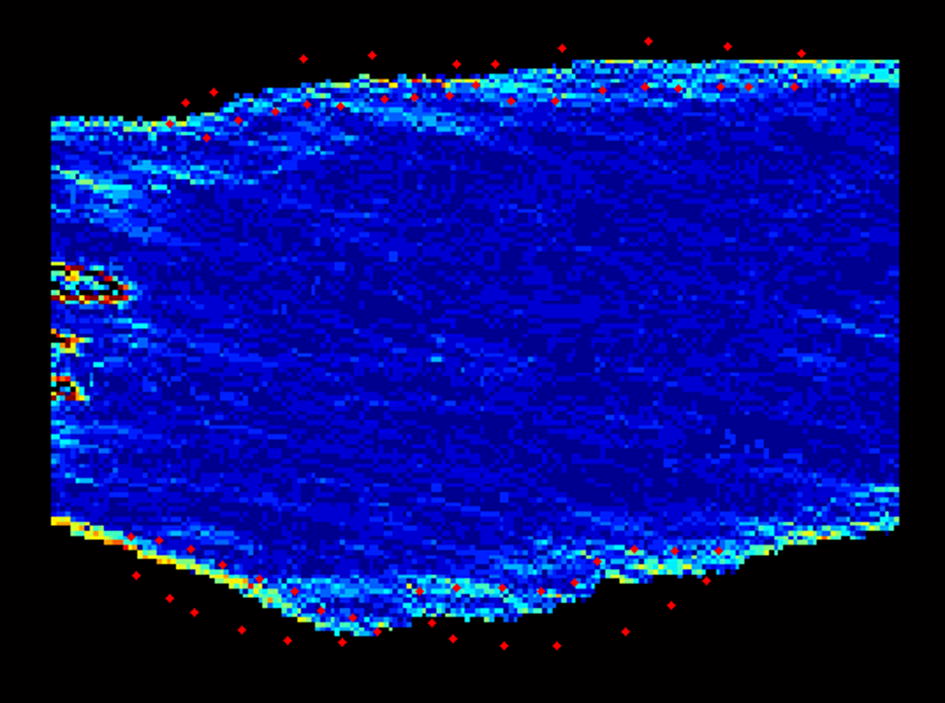
Two-dimensional spatial distribution of WSS sampling point in the carotid sinus

Three long axis dynamic images of the CCA in 3 cardiac cycles were obtained, including the distal end of CCA (about 2 cm below the CCA bifurcation), the carotid sinus (between the end of the CCA bifurcation and the beginning of the ICA), the proximal end of the ICA. All CDFI images were saved in DICOM (image matrix, 600 × 800; pixel spacing, 0.085 × 0.085 mm) format and imported offline to the WSS quantitative analysis software by the MATLAB (The Mathworks Inc. Natick, MA, USA) platform to analyze. Then, the WSS of each pixel in CDFI, the two- and three-dimensional spatial distribution WSS maps and a fused WSS image were obtained as shown in Fig. 1. In this figure, A1 and B1 pertains to the CDFI of Carotid Sinus and the fusion image of WSS distribution with ultrasonic image. A2 and B2 were the segmented CDFI of Carotid Sinus and the two-dimensional distribution of WSS. A3 and B3 were the three-dimensional distribution of blood velocity profile in Carotid Sinus and the three-dimensional distribution of WSS. WSS calculated equation was $\tau_{w} = \mu \frac{du}{dr} |r = wall$, with the assumption that blood is a Newtonian fluid. In this equation, $\tau_{w}$ is WSS, which is the shear stress near the vessel wall. *μ* is blood viscosity. $\frac{du}{dr}$ is velocity gradient (or shear rate). $r = wall$ means that *r* is near the boundary of tube. Detailed WSS quantitative analysis steps were reported in our previous study [18]. Based on obtaining the spatial distribution of the two-dimensional WSS imaging, we then collected six regional locations, namely posterior wall of CCA, posterior wall of ICA, posterior-interior wall of the proximal carotid sinus, posterior-interior wall of the distal carotid sinus, the anterior-lateral wall of the proximal carotid sinus, as well as the anterior-lateral wall of the distal carotid sinus. The sampling point is shown in Fig. 2. The average WSS of carotid sinus was computed based on the above four values. After recording, the software can automatically import the Excel form in the background and calculate the WSS distribution range and average value of each area of interest.

#### Statistical analysis

All WSS data were analyzed by Microsoft Excel and SPSS 22.0 software (SPSS Inc., Chicago, IL, USA). Shapiro–Wilk test was used to assess the distribution of continuous variables. Results were expressed as frequencies and percentages for categorical variables, mean ± standard deviation (SD) for normally distributed variables and median (interquartile range) for non-normally distributed variables. Comparisons between continuous variables were performed using Independent-Samples T-test, Mann–Whitney U-test, One Way ANOVA test or Kruskal–Wallis test as appropriate. Chi square test was used for comparison of categorial variables. Correlations between the IMT and local WSS of carotid sinus was verified by Pearson correlation coefficients. P < 0.05 is considered significant for all described analyses.

## Result

### Clinical parameters

The general clinical indexes of the IMT normal group and the IMT thickening group are summarized in Table 1. There was no significant difference in age, sex, height, weight and heart rate between the two groups (P > 0.05). However, the cases of hypertension, coronary heart disease, diabetes, hyperlipidemia and smoking were statistically different (P < 0.05).

**Table 1:** Clinical characteristics in the IMT normal group and the IMT thickening group

Variable	The IMT normal group, n = 100	The IMT thickening group, n = 50	P value
Age (years)	55.68 ± 13.46	69.12 ± 11.62	0.951
Sex (male/female)	44/56	28/22	0.166
Height (cm)	170.47 ± 6.32	173.25 ± 7.11	0.084
Weight (kg)	65.32 ± 15.87	70.39 ± 16.45	0.072
Heart rate (bpm)	73.25 ± 7.14	68.58 ± 6.22	0.545
Hypertension (yes/no)	32/68	42/8	0.000*
CHD (yes/no)	14/86	32/28	0.000*
Diabetes (yes/no)	4/96	12/38	0.000*
Hyperlipidemia (yes/no)	5/95	18/32	0.000*
Smoking (yes/no)	22/78	24/26	0.001*

*CHD* coronary heart disease

* P < 0.05

### Conventional parameters of carotid ultrasound

The conventional carotid ultrasound parameters of the two groups were compared in Table 2. There was no significant difference in the internal diameter of CCA, PSV and RI between the two groups. The conventional WSS of CCA in IMT thickening group was lower than that in normal group.

**Table 2:** Conventional carotid ultrasound parameters in the IMT normal group and the IMT thickening group

Variable	IMT normal group, n = 100	IMT thickening group, n = 50	P value
CCA inner diameter (mm)	6.91 ± 0.5	6.73 ± 0.4	0.257
CCA IMT (mm)	0.69 ± 0.16	0.99 ± 0.19	0.000*
PSV (cm/s)	91.3 ± 20.7	95.4 ± 18.8	0.188
RI	0.72 ± 0.05	0.69 ± 0.08	0.358
WSS of CCA^a^	7.81 ± 2.72	6.94 ± 1.38	0.034*

* P < 0.05

^a^Computed by Hagen–Poiseuille formula

### WSS quantitative analysis

#### Respective WSS spatial distribution in two group

**Fig. 3:**
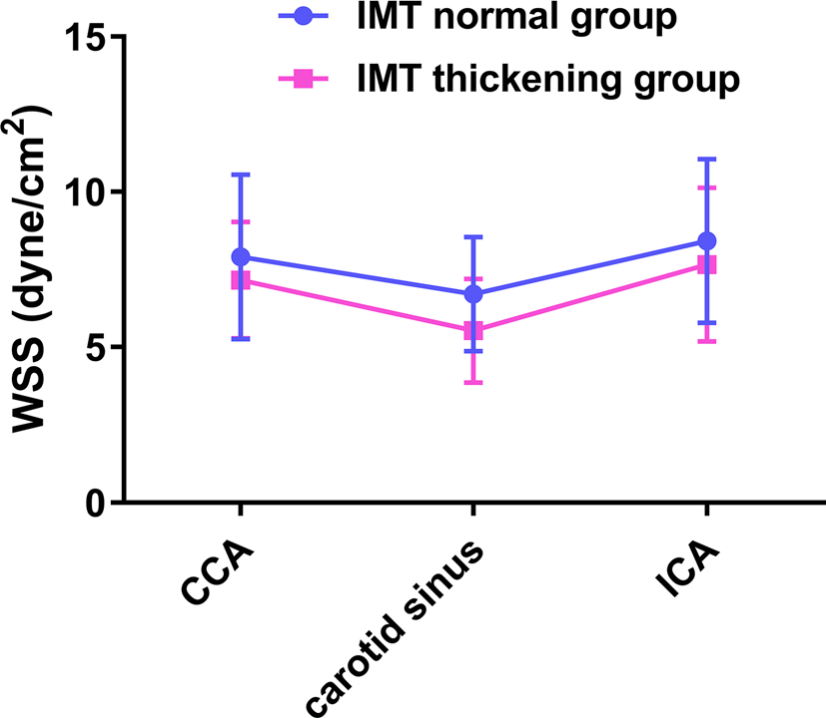
WSS spatial distribution in two groups

As shown in Fig. 3, we can preliminarily find the WSS of carotid sinus < CCA < ICA in both IMT normal group and IMT thickening group.

**Table 3:** WSS in the IMT normal group and the IMT thickening group

WSS (dyne/cm^2^)	IMT normal group, n = 100	IMT thickening group, n = 50	P value
CCA	7.91 ± 2.65	7.15 ± 1.88	0.027*
ICA	8.42 ± 2.64	7.65 ± 2.48	0.031*
ALPW of carotid sinus	6.02 ± 1.79	5.08 ± 1.06	0.025*
ALDW of carotid sinus	5.98 ± 2.01	5.33 ± 1.56	0.001*
PIPW of carotid sinus	7.52 ± 2.36	5.86 ± 1.76	0.001*
PIDW of carotid sinus	7.31 ± 1.25	5.85 ± 2.04	0.034*

*ALPW* the anterior-lateral proximal wall, *ALDW* the anterior-lateral distal wall, *PIPW* the posterior-interior proximal wall, *PIDW* the posterior-interior distal wall

* P < 0.05

**Fig. 4:**
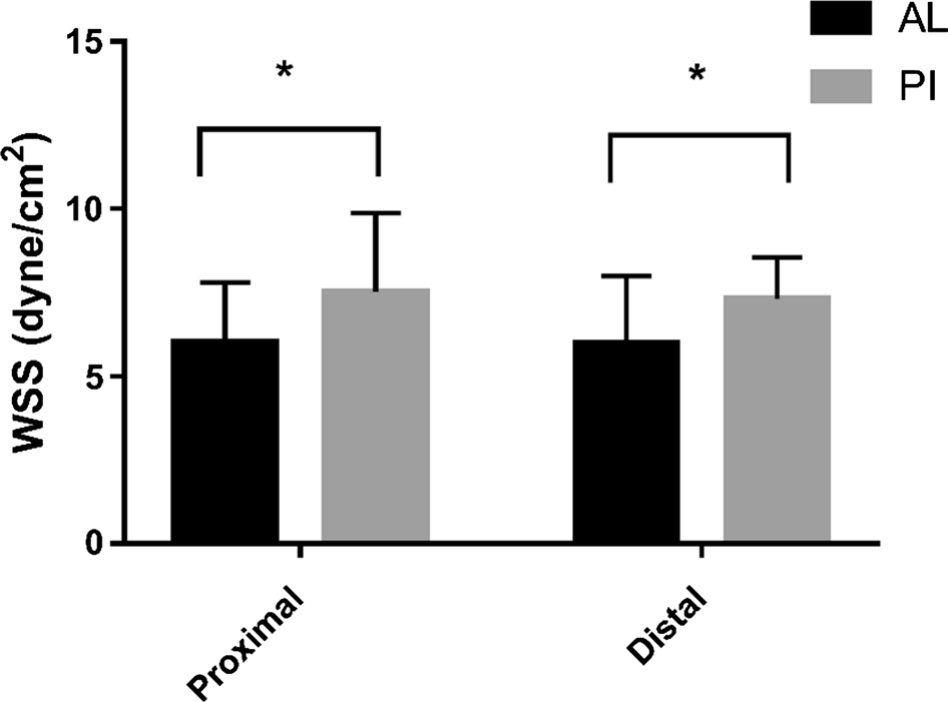
WSS of carotid sinus in IMT normal group. * P < 0.05; *AL* the anterior-lateral, *PI* the posterior-interior

**Fig. 5:**
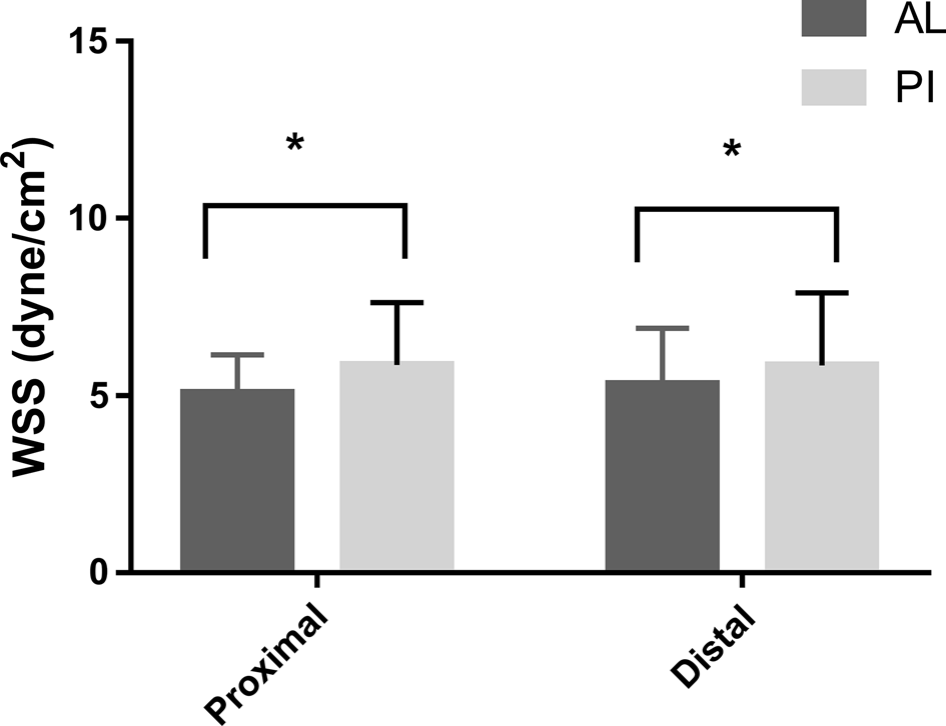
WSS of carotid sinus in IMT thickening group. * P < 0.05; *AL* the anterior-lateral, *PI* the posterior-interior

As shown in Figs. 4 and 5, there was no significant difference between the proximal and distal ends of the carotid sinus (P > 0.05), while the anterior-lateral wall of carotid sinus < posterior-interior wall in both two group (P < 0.05).

#### Spatial distribution of WSS between IMT thickening group and normal group

Comparing the two groups (Table 3), we found that the WSS of CCA, ICA, proximal and distal ends of the anterolateral wall, proximal and distal ends of the posterolateral wall of the carotid sinus in IMT thickening group were lower than those of IMT normal group (P < 0.05).

#### The correlation between the IMT and local WSS of carotid sinus

On account of 4 regional locations in each carotid sinus, there were total 200 locations in IMT thickening group, among which 122 IMT thickening locations and 78 IMT normal locations. Furthermore, the WSS of the former locations was smaller than the latter (P < 0.05), as shown in Table 4. Then a bivariate correlation analysis between the IMT of 200 locations corresponding to WSS results in correlated coefficient r = − 0.352 (P = 0.000), showing a negative weak correlation.

**Table 4:** WSS in IMT thickening regions and IMT normal regions of carotid sinus

Carotid sinus	Numbers	WSS	P value
IMT thickening location	122	4.18 ± 1.35	0.035*
IMT normal location	78	7.93 ± 2.07	

* P < 0.05

## Discussion

### Traditional risk factors for atherosclerosis

Atherosclerosis remains the leading cause of death and morbidity worldwide. Noninvasive imaging techniques such as ultrasound are currently available for the detection of subclinical atherosclerosis and assessment of its progression and regression [19]. Previous studies had proved that hypertension, dyslipidemia, diabetes, obesity and smoking are some traditional risk factors for cardio-cerebrovascular diseases [20]. By comparison with the clinical data between the IMT thickening group and the normal group, we found that the incidence of hypertension, coronary heart disease, diabetes, hyperlipidemia and smoking in the IMT thickening group are higher than the IMT normal group, which also proved that these diseases are closely related to atherosclerosis. However, such traditional risk factors only reflected the susceptibility of atherosclerosis and could not demonstrate the spatial distribution and dynamic development of atherosclerosis in individuals. Therefore, we need to find some other sensitive and accurate index to predict the potential development of atherosclerosis and plaque formation at early stage.

#### Evaluation indexes and methods of atherosclerosis

The most common clinical detection of atherosclerosis is to measure IMT by using ultrasonic imaging. The internal diameter of CCA, IMT, PSV and RI were recorded by conventional vascular ultrasound, and the conventional WSS was estimated by traditional formula Hagen–Poiseuille, $\tau_{w} = \frac{{2\mu u_{m} }}{R}$. There was no significant difference in internal diameter of CCA, PSV and RI between the two groups (P > 0.05), but the WSS of CCA $\tau_{w}$ in IMT thickening group was significantly lower (P < 0.05). We can conclude that the conventional parameters from carotid ultrasound are not sensitive in detecting atherosclerosis. The possible reason is that atherosclerosis is a long-term process from quantitative accumulation to qualitative transformation [5, 6]. When the vascular wall at the early stage of IMT thickening, there is no obvious changes in vascular morphology or regional hemodynamics [21]. In contrast to these conventional evaluation indexes, the WSS calculated by formula Hagen–Poiseuille showed a difference between the IMT thickening group and the IMT normal group. This suggests that WSS may be a sensitive indication for early evaluation of atherosclerosis, which is consistent with previous studies on the relationship between WSS and atherosclerosis mentioned in the background [22]. According to the principle of Hagen–Poiseuille formula, the equation is based on the diameter of the vessel and the maximum flow velocity and represents the value of WSS of all points in the cross section of the vascular wall [23]. However, the arterial vessel of human is not likely to be standard round, and the WSS will be affected by different factors such as blood flow, blood pressure, geometric structure of the vessel wall, IMT, etc., which will result in different WSS values at each point of the vessel wall [24]. Therefore, the results calculated by the Hagen–Poiseuille formula can only be used as rough estimates, which cannot reflect the local WSS changes near the vascular wall. In addition, the Hagen–Poiseuille formula were mainly used to estimate large arteries with cylindrical tube and rigid wall such as CCA. It is impossible to accurately evaluate the WSS of irregular blood vessel such as carotid sinus with a local intumescentia [25]. The WSS quantitative analysis framework that is used in this study is developed independently and based on MATLAB platform. Our software makes use of digital image processing technology to extract the blood flow signal from CDFI. The color data of each pixel in CDFI is converted into blood flow velocity data and then converted into shear stress data according to the definition formula of WSS. Theoretically, the WSS distribution of blood vessels with any shape can be analyzed. In previous study, the design principle of the WSS quantitative analysis software was expounded and it was used to measure the spatial distribution of WSS in the normal CCA in vivo [18]. Zhang et al. compared the accuracy of WSS to Hagen–Poiseuille formula, which shows that this method can hopefully present more accurate and detailed values of regional WSS near vessel walls [26].

#### Hemodynamic changes at carotid sinus

Carotid sinus is one special bifurcation of carotid artery with complex structure and individual geometry, where hemodynamics changes suddenly and the blood flow is turbulent [27]. On account of the blood flow shunting to ICA and ECA, the WSS of carotid sinus altered. Previous studies have found that the inner wall of two sub-vessels in the of the carotid bifurcation divides the blood flow into two tributaries, and the corresponding wall is subjected to the largest axial impact forming a high shear stress region [28]. Owning to the blood flow of the carotid bifurcation flowing into the sub-vessels at a certain angle, so the lateral wall of branch cavity inlet is turbulent due to the flow separation and then forms a low WSS region [29]. But in the past studies, most WSS data were acquired from artificial models, so the observed data in vivo was not enough. This study is based on CDFI to evaluate the distribution of WSS and changing regularities in carotid sinus in vivo. Hemodynamic changes occurring at the initial segments of the arterial bifurcations play a vital role in the development of atherosclerotic plaque, so atherosclerotic plaque is likely to locate in carotid sinus [30]. So under such condition of noninvasive methodology, the self-developed WSS quantitative analysis software combined with texture matching method can detect the early diagnosis of atherosclerosis and can obtain specific WSS for further evaluation.

### Limitations and future developments

Firstly, the WSS data from CDFI in our study are not three-dimensional data as compared to MRA. However, ultrasound scanning is simple, time-saving and relatively economical compared with MRA, which makes WSS assessment easier to apply. In addition, our CDFI-based program cannot be applied for detecting the WSS of the coronary artery, which can be further assessed by angiography or intravascular ultrasound. At last, the outcome in IMT thickening group was not been long-term followed. Therefore, a large long-term followed study subject group is required to confirm the development of atherosclerosis.

## Conclusion

In summary, WSS of IMT thickening location is becoming lower in early stage of atherosclerosis. This variation was showed in CCA, ICA, and all parts of the carotid sinus. The distribution of WSS whether in IMT normal group or IMT thickening group was approximately as follows: carotid sinus WSS < WSS of CCA < WSS of ICA, and WSS of anterior-lateral wall of carotid sinus < WSS of posterior-interior wall. The internal distribution was stable. Therefore, it provides a useful tool for early and accurate prediction of the site of atherosclerosis in clinical.
